# Associations of maternal nutrition during pregnancy and post‐partum with maternal cognition and caregiving

**DOI:** 10.1111/mcn.12546

**Published:** 2017-11-02

**Authors:** Elizabeth L. Prado, Ulla Ashorn, John Phuka, Kenneth Maleta, John Sadalaki, Brietta M. Oaks, Marjorie Haskell, Lindsay H. Allen, Steve A. Vosti, Per Ashorn, Kathryn G. Dewey

**Affiliations:** ^1^ Department of Nutrition University of California Davis Davis California USA; ^2^ Center for Child Health Research University of Tampere School of Medicine and Tampere University Hospital Tampere Finland; ^3^ School of Public Health and Family Medicine University of Malawi College of Medicine Blantyre Malawi; ^4^ USDA, ARS Western Human Nutrition Research Center University of California Davis Davis California USA; ^5^ Department of Agricultural and Resource Economics University of California Davis Davis California USA; ^6^ Department of Paediatrics Tampere University Hospital Tampere Finland

**Keywords:** caregiving, DHA, iLiNS Project, lipid‐based nutrient supplements, maternal cognition, Vitamin B12

## Abstract

Pregnant and post‐partum women require increased nutrient intake and optimal cognition, which depends on adequate nutrition, to enable reasoning and learning for caregiving. We aimed to assess (a) differences in maternal cognition and caregiving between women in Malawi who received different nutritional supplements, (b) 14 effect modifiers, and (c) associations of cognition and caregiving with biomarkers of iron, Vitamin A, B‐vitamin, and fatty acid status. In a randomized controlled trial (*n* = 869), pregnant women daily received either multiple micronutrients (MMN), 20 g/day lipid‐based nutrient supplements (LNS), or a control iron/folic acid (IFA) tablet. After delivery, supplementation continued in the MMN and LNS arms, and the IFA control group received placebo until 6 months post‐partum, when cognition (*n* = 712), caregiving behaviour (*n* = 669), and biomarkers of nutritional status (*n* = 283) were assessed. In the full group, only one difference was significant: the IFA arm scored 0.22 *SD* (95% CI [0.01, 0.39], *p* = .03) higher than the LNS arm in mental rotation. Among subgroups of women with baseline low hemoglobin, poor iron status, or malaria, those who received LNS scored 0.4 to 0.7 *SD* higher than the IFA arm in verbal fluency. Breastmilk docosahexaenoic acid and Vitamin B12 concentrations were positively associated with verbal fluency and digit span forward (adjusting for covariates *p*s < .05). In this population in Malawi, maternal supplementation with MMN or LNS did not positively affect maternal cognition or caregiving. Maternal docosahexaenoic acid and B12 status may be important for post‐partum attention and executive function.

## 1 INTRODUCTION

Brain function depends on a continuous supply of nutrients, including macronutrients, micronutrients, and fatty acids. Most studies investigating the effects of nutritional supplementation on cognitive function have focused on children and the elderly, due to the increased nutrient demands and likelihood of deficiency plus the importance of optimizing cognitive function during these periods of the lifespan. Pregnancy and post‐partum are also periods of increased nutrient demands, and many women in low‐ and middle‐income countries are at risk for nutrient deficiencies during these periods (Black et al., 2013). Optimal maternal cognition is important for reasoning, decision‐making, and learning new information to prepare for a healthy birth and to care for a young infant. Higher maternal cognitive performance, independent of education level, is associated with a lower risk of infant mortality (Sandiford, Cassel, Sanchez, & Coldham, 1997), malnutrition (Anoop, Saravanan, Joseph, Cherian, & Jacob, 2004), and poor cognitive development (Tong, Baghurst, Vimpani, & McMichael, 2007). Although some interventions focus on promoting health knowledge, the possibility of improving infant health and development by enhancing maternal cognitive skills essential for effective caregiving, for example, through nutritional supplementation, has received little attention.

Few studies of the effects of maternal nutritional supplementation on maternal cognition and caregiving have been conducted. Among four studies that examined nutrition and cognition during pregnancy and post‐partum (Beard et al., 2005; Groner, Holtzman, Charney, & Mellits, 1986; Prado, Ullman, Muadz, Alcock, & Shankar, 2012; Stoecker et al., 2009), three were randomized trials (Beard et al., 2005; Groner et al., 1986; Prado et al., 2012), but only one included a sample of greater than 100 participants (Prado et al., 2012). All of these studies focused on micronutrients without examining other nutrients important for brain function, such as fatty acids, and no study included cognitive tests directly relevant to caregiving. Five studies that have investigated maternal nutrition and mother–child interaction have similar limitations of small sample sizes (maximum 180) and focus on micronutrients without fatty acids (Frith, Naved, Ekstrom, Rasmussen, & Frongillo, 2009; Kirksey et al., 1992; McCullough et al., 1990; Murray‐Kolb & Beard, 2009; Wachs et al., 1992).

In the current study, we address these gaps using data from >700 women who participated in the International Lipid‐Based Nutrient Supplements DYAD‐M Project in Malawi. We assessed cognitive skills tied to brain systems likely to be affected by nutrient deficiency, as well as functional health literacy, which requires cognitive skills such as memory and comprehension of words and pictures and is more directly relevant to caregiving. For example, a mother must understand written medication instructions, or remember verbal instructions from a doctor, in order to provide the correct dose to her child. We examined supplementation not only with iron and other micronutrients but also with essential fatty acids, specifically the omega‐3 fatty acid alpha‐linolenic acid (ALA), a precursor to docosahexaenoic acid (DHA), and the omega‐6 fatty acid linoleic acid, a precursor to arachidonic acid (AA). Both DHA and AA are structural components of cerebral membranes, affecting their enzymatic activities, binding between molecules and receptors, cellular interactions, and nutrient transport (Bourre, 2004).

The main objective of the current study was to assess the differences in maternal cognitive performance and caregiving at 6 months post‐partum between three groups of women who received different nutritional supplements over a 1‐year period from ≤20 weeks gestation to 6 months post‐partum. Our hypothesis was that women who received multiple micronutrients (MMN) or lipid‐based nutrient supplements (LNS) would score higher than a control group that received iron and folic acid (IFA). We tested whether initial nutritional status modified the effects of the intervention (maternal iron status, hemoglobin, height, and body mass index [BMI]), as well as additional prespecified effect modifiers: baseline malaria, education, age, gestational age, primiparity, season at enrollment, study site, household food insecurity access scale score, and household asset index. The second hypothesis was that maternal scores would be associated with concurrent nutritional status, measured by biomarkers of iron, Vitamin A, B‐vitamins, and DHA in a subgroup of 283 women.

**Key messages**

- Breastmilk docosahexaenoic acid and Vitamin B12 concentrations were associated with attention and executive function in post‐partum women in Malawi.
- Maternal supplementation during pregnancy and post‐partum with multiple micronutrients or lipid‐based nutrient supplements, compared to iron and folic acid, did not positively affect these or other aspects of cognition or caregiving, as measured in this study.
- The association of lipid‐based nutrient supplements with higher verbal fluency scores in subgroups of women with baseline low hemoglobin, poor iron status, or malaria suggests that further research on the cognitive effects of supplementation targeting such women is needed.

## 2 METHODS

### 2.1 Study participants and design

We conducted an add‐on study of maternal cognition and caregiving in a randomized trial described in more detail by Ashorn et al. (Ashorn et al., 2015), designed to assess the effect of maternal and infant LNS on infant growth. Pregnant women (*n* = 869) who attended antenatal care at two hospitals and one health center in Mangochi district, Malawi, were enrolled from February 2011 to March 2012 and assigned to one of three intervention arms, described below. Details of randomization and inclusion criteria have been published previously (Ashorn et al., 2015). All participants provided informed consent. Ethical approval for the study procedures was obtained from the Ethics Committees at the University of Malawi College of Medicine and Tampere University Hospital District, Finland. The study was registered with the U.S. National Institutes of Health as a clinical trial (http://www.clinicaltrials.gov; NCT01239693). The sample size of 290 per group, allowing for 20% attrition, provided 83% power to detect a difference of 0.3 *SD* in continuous scores between groups, with alpha at 0.05 (Zhao & Li, 2012).

At enrollment, which occurred at ≤20 weeks gestation, women were randomly assigned to one of three intervention arms; all of whom received two doses of intermittent preventive malaria treatment during pregnancy. The IFA arm received standard antenatal care, including supplementation from enrollment to delivery with one capsule per day containing 60 mg iron and 400 μg folic acid. The IFA arm received a placebo tablet containing 200 mg calcium from delivery to 6 months post‐partum. The MMN arm received one capsule per day from enrollment to 6 months post‐partum that contained a lower dose of iron (20 mg), 400 μg folic acid, and 16 additional micronutrients, shown in Table 1. The LNS arm received daily 20‐g sachets of small quantity LNS produced by Nutriset SAS (Malaunay, France) from enrollment to 6 months post‐partum, containing the same micronutrients as the MMN capsules, four additional minerals, protein, fat, and essential fatty acids (Table 1).

**Table 1:** Nutrient and energy contents of the dietary supplements

	IFA^a^	MMN^b^	LNS^b^
Ration per day	1 tablet	1 tablet	20‐g sachet
Total energy, kcal	0	0	118
Protein, g	0	0	2.6
Fat, g	0	0	10
Linoleic acid, g	0	0	4.59
α‐Linolenic acid, g	0	0	0.59
Vitamin A, mg RE	0	800	800
Vitamin C, mg	0	100	100
Vitamin B‐1, mg	0	2.8	2.8
Vitamin B‐2, mg	0	2.8	2.8
Niacin, mg	0	36	36
Folic acid, μg	400	400	400
Pantothenic acid, mg	0	7	7
Vitamin B‐6, mg	0	3.8	3.8
Vitamin B‐12, mg	0	5.2	5.2
Vitamin D, mg	0	10	10
Vitamin E, mg	0	20	20
Vitamin K, mg	0	45	45
Iron, mg	60	20	20
Zinc, mg	0	30	30
Copper, mg	0	4	4
Calcium, mg	0	0	280
Phosphorus, mg	0	0	190
Potassium, mg	0	0	200
Magnesium, mg	0	0	65
Selenium, mg	0	130	130
Iodine, mg	0	250	250
Manganese, mg	0	2.6	2.6

*Note*. IFA = iron/folic acid; MMN = multiple micronutrients; LNS = lipid‐based nutrient supplements.

a The group who received IFA from enrollment to delivery received placebo (200 mg/day calcium) from delivery to 6 months post‐partum.

b MMN and LNS were provided from enrollment to 6 months post‐partum.

The iron dose was lower for participants in the MMN and LNS arms (20 mg/day) than for those in the IFA arm (60 mg/day), because supplementation with MMN and LNS continued during the first 6 months post‐partum, when the recommended iron intake for lactating women is much lower than the standard antenatal dose (Arimond et al., 2015). On the basis of a literature review and our estimates of the normal dietary iron intakes among pregnant women in the study area, we considered 20 mg/day a safe and adequate dose to prevent iron deficiency anemia during pregnancy, even for women who were iron deficient at enrollment (Arimond et al., 2015).

At the time of enrollment, data collectors recorded sociodemographic and maternal anthropometric data. Research nurses assessed the duration of pregnancy with ultrasonography and measured the women's peripheral blood malaria parasitemia and HIV infection with rapid tests. Maternal hemoglobin concentration (Hb; g/dl) was determined using on‐site cuvette readers (HemoCue AB; Angelholm), and zinc protoporphyrin concentration (ZPP; μmol/mol heme) was determined from venous blood samples using a hematofluorometer (Aviv Biomedical Co. NJ, USA), after red blood cells were washed three times with normal saline. Plasma soluble transferrin receptor (sTfR; mg/L) was determined from plasma by immunoturbidimetry on the Cobas Integra 400 system autoanalyzer (F. Hoffmann‐La Roche Ltd, Basel, Switzerland). We determined cut‐off values for low Hb, indicating anemia, and elevated ZPP and sTfR, indicating low iron status, following Adu‐Afarwuah et al. (Adu‐Afarwuah et al., 2016): Hb < 100 g/L, ZPP > 70 μmol/mol heme, and sTfR > 6 mg/L. Research staff delivered supplements to participants' homes every 2 weeks and collected remaining supplements. Adherence was calculated as the percent of delivered supplements that were not returned to research staff. For a detailed description of these variables, see Ashorn et al (Ashorn et al., 2015).

### 2.2 Assessment of biomarkers of nutritional status at 6 months post‐partum

Maternal Hb and ZPP were assessed during a clinic visit at 6 months post‐partum in the same way as described above. A subsample of 369 women was randomly selected for assessment of additional biomarkers of nutritional status. Plasma retinol concentration (μmol/L) was assessed by high‐performance liquid chromatography, as previously described (Bieri, Tolliver, & Catignani, 1979). Breastmilk samples were collected at a home visit. Breastmilk DHA (percentage by weight of total fatty acids) was assessed by gas chromatography with flame ionization detection using a GC‐2010 (Shimadzu Corporation, Columbia, MD) equipped with a SP‐2560, 100‐m fused silica capillary column (Supelco, Bellefonte, PA; Oaks et al., 2017). Breastmilk concentrations of Vitamins B1, B2, B3, B6, and B12 were assessed at the Western Human Nutrition Research Center. Free thiamin, thiamin monophosphate, and thiamin triphosphate were measured by high‐performance liquid chromatography‐fluorescence detection after precolumn derivatization to their thiochrome esters (Hampel et al., 2016). Thiamin (B1) was calculated as free thiamin + (thiamin monophosphate × 0.871) + (thiamin pyrophosphate × 0.707). Riboflavin (B2), nicotinamide (B3), pyridoxal (B6), and flavin adenine dinucleotide were measured by UPLC‐MS/MS (Waters, Milford, MA coupled to 4000 QTRAP LC‐MS/MS, AB Sciex, Foster City, CA) as previously described (Hampel, York, & Allen, 2012). Riboflavin was calculated as free riboflavin + (flavin adenine dinucleotide × 0.479). Vitamin B12 was analyzed using an IMMULITE® E‐411 competitive binding assay (Duluth, GA, USA) as previously described (Hampel et al., 2014). For further details, see Supplemental Methods.

### 2.3 Assessment of maternal cognition and caregiving

A team of five project staff, who were blind to intervention arm, visited participants at their homes at 6 months post‐partum to assess maternal cognition and caregiving. If family members or neighbors gathered to observe the assessments, data collectors politely asked them to leave in order to create a private environment, which was generally successful. Apart from the participants, one or more other adults were present at 5% of the visits, and one or more other children were present at 12% of the visits.

To assess cognition, we selected three tests that were previously adapted for use in a maternal supplementation trial in Indonesia (Prado et al., 2012). In that study, tests were selected on the basis of the following criteria: widely‐used tests that primarily tap aspects of specific cognitive functions; are tied to particular brain structures and mechanisms; may be affected by micronutrient deficiency based on previous studies; do not require special equipment; do not require literacy; are easily administered and scored; and do not require subjective judgments from the testers. For this study, of the six cognitive tests used by Prado et al. (2012), we selected the three that did not require verbal stimuli to be developed in the local languages: digit span forward and backward, category fluency, and mental rotation.

Digit span forward and backward tests measure attention, verbal short‐term memory, and working memory, which are rooted areas in the right dorsolateral prefrontal cortex and bilateral inferior parietal lobule, as well as the anterior cingulate (Gerton et al., 2004). Participants were orally presented with increasingly longer sequences of digits and instructed to either repeat them (digit span forward) or repeat them backwards (digit span backward), until an error was committed on two consecutive trials. The score was the number of sequences correctly repeated.

Category fluency assesses semantic memory, which is rooted in areas of the temporal lobe, and executive function, which is rooted in areas of the frontal lobe (Birn et al., 2010). Participants were asked to name as many members of a category as possible in 1 min, first for the category “food” and second for the category “girl's names.” The score was the average of the two trials.

Mental rotation measures visuospatial ability and dynamic mental imagery and activates areas in the superior parietal cortex, inferior prefrontal cortex, and other structures (Zacks, 2008). The participant was visually presented with five rows of figures and instructed to mark the figures that were rotations but not mirror images of the target figure. The score was the percent correct.

All cognitive tests were audio recorded and reviewed by a data collector who did not conduct the assessment, to correct any errors. The supervisor reviewed 10% of each batch of submitted forms and recordings. If any error was found, the supervisor reviewed the entire batch and corrected any errors. Thirty‐two participants were tested twice to evaluate test–retest reliability, with a mean test–retest interval of 7 days. Test–retest reliability (Pearson's *r*) was 0.62 for digit span forward, 0.50 for digit span backward, 0.66 for category fluency, and 0.63 for mental rotation.

We developed a functional health literacy test to assess memory and understanding of health messages communicated in words and pictures, on the basis of a test developed in Ethiopia (Stevenson, 2011). We selected health materials that were common in Malawi and that were relevant for children's health, such as medication instructions, breastfeeding information, and growth charts. The score was the number of questions answered correctly out of a maximum 36 points. Test–retest reliability was 0.78. Because this test was not audio recorded, we also assessed inter‐rater agreement by periodically assigning pairs of data collectors to visit 32 women. One person conducted the test whereas the other independently completed the form. Inter‐rater agreement was 94%.

We assessed maternal caregiving behavior using the Infant/Toddler version of the Home Observation for the Measurement of the Environment (HOME) Inventory (Caldwell & Bradley, 2003). The HOME Inventory measures the amount and quality of stimulation that children receive from their environment. Items assess maternal responsivity, acceptance, and involvement as well as the learning materials, variety, and organization in the child's environment. To adapt the items to the local context, we conducted a focus group discussion with 12 mothers of young children in each of the three study sites. We used this information to add locally‐appropriate examples, such as toys for making music, and to modify items to increase variance in scores. For example, we changed “At least ten books are visible in the home” to “At least one book is visible in the home.” We eliminated nine items for which we could not find an appropriate modification (e.g., “Child has a special place for toys and treasures”). The adapted tool comprised 18 items coded by observation, 12 by interview, and 6 by observation or interview, according to the standard procedure. The total score was the sum of the item scores, each of which was scored 0 or 1. Inter‐rater agreement was 89% and test–retest reliability was 0.82, using the same procedures described above. The HOME score showed expected correlations with household asset index (*r* = 0.29, *p* < .001) and maternal education (*r* = 0.27, *p* < .001), providing evidence for convergent validity.

### 2.4 Statistical analyses

All analyses were conducted using SAS Version 9.4 (SAS Institute, Cary, NC). The primary analysis was by intention to treat. We also conducted per protocol analyses excluding women with less than 80% adherence to supplement consumption.

For each score, we computed *z*‐scores on the basis of the distribution of our sample. All cognitive scores were normally distributed, after truncating outliers to the 1st and 99th percentile (2 digit span forward scores, 7 digit span backward scores, and 3 HOME scores). We estimated the difference between the intervention arms first in unadjusted models using analysis of variance and then in adjusted models using analysis of covariance. For each outcome, we determined a set of covariates as any of 17 prespecified covariates that independently predicted that outcome score at *p* < .1. All potential covariates are listed in Footnote 2 of Table 3. If the *F* value was significant at *p* < .05, we used Tukey–Kramer's test to adjust for multiple comparisons for pairwise comparisons between groups.

As potential effect modifiers, we examined baseline ZPP and sTfR, plus 12 additional effect modifiers defined a priori: baseline maternal height, BMI, Hb, malaria, education, age, and gestational age; primiparity, season at enrollment, and study site; and household food insecurity access scale score and household asset index. If any interaction between the potential effect modifier and intervention arm was significant at *p* < .05, we further explored the pattern of the effect at high and low levels of the effect modifier.

To eliminate skewness in the biomarker variables, we applied the following transformations: log transformation for ZPP and breastmilk DHA; square root transformation for breastmilk Vitamin B3, B6, and B12; and inverse transformation for breastmilk Vitamin B2. We then entered all nine biomarkers collected at 6 months post‐partum into a principal component analysis (PCA) to reduce the number of variables examined. We retained three components with eigenvalues >1. The first component represented higher nutritional status on all variables, with all eigenvectors >0.3 except breastmilk B1 (0.19), plasma retinol (0.18), and breastmilk DHA (0.05). The second component represented higher breastmilk B1, B2, and B6, but lower Hb, iron, and Vitamin A status. The third component represented higher breastmilk DHA (eigenvector = 0.51) and Vitamin B12 (eigenvector = 0.59). For details, see Table S1. For each maternal cognitive and caregiving score, we examined the association with each of the three nutritional biomarker components, adjusting for the same set of covariates specific to that outcome.

## 3 RESULTS

**Figure 1:**
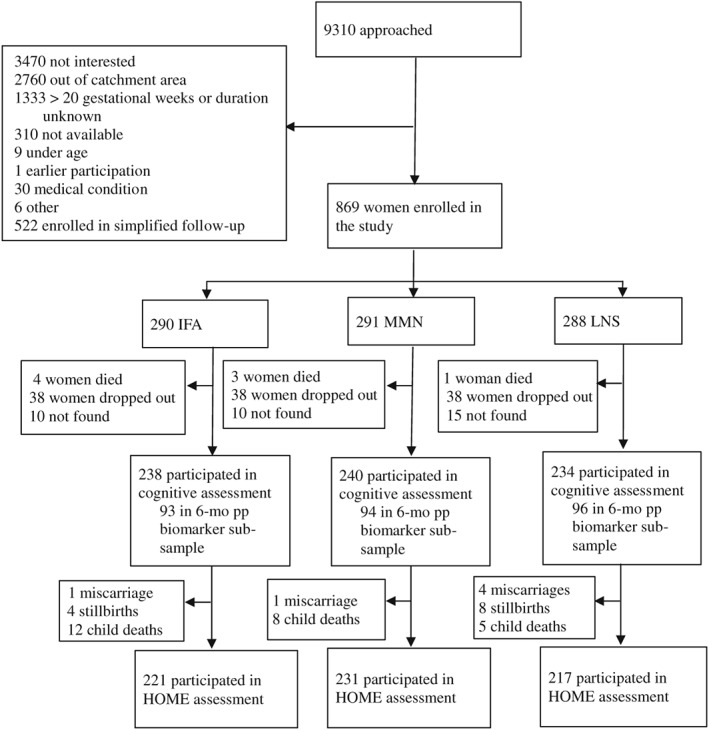
Trial Profile. IFA = iron/folic acid; MMN = multiple micronutrients; LNS = lipid‐based nutrient supplements; HOME = home observation for the measurement of the environment

Figure 1 shows the trial profile. Out of 869 women enrolled, 712 (82%) participated in cognitive assessment, and 669 (77%) participated in HOME assessment. The proportion of participants who were not assessed was not significantly different between the three intervention arms (cognitive assessment: chi‐square = 0.15; *p* = .93; HOME: chi‐square = 1.53; *p* = .47). The mean amount of time between enrollment and delivery was 22 (*SD* = 4) weeks and between delivery and cognitive testing was 30 (*SD* = 6) weeks. Of the 369 women randomly selected for the biomarker subsample, complete data for all nine biomarkers were available from 283 (Figure 1).

Table 2 presents baseline characteristics of the cognitive sample in each intervention arm and the characteristics of the women who were enrolled but did not participate in cognitive assessment. Women in the three intervention arms did not differ significantly in any of the baseline characteristics. A large proportion of the women who did not participate in cognitive assessment (72%) had enrolled at the public hospital in Mangochi, which served a more transient population than the other two sites at Malindi and Lungwena. Compared to those who did participate in cognitive assessment, enrolled women who did not participate were significantly younger and had higher education and BMI. A greater proportion were primiparous, and a smaller proportion had a household asset index below the median (Table 2).

**Table 2:** Baseline characteristics of each intervention group, the sample who participated in cognitive assessment, and those lost to follow‐up

	CS‐IFA	CS‐MMN	CS‐LNS	*p*‐value^a^	CS	Non‐CS	*p*‐value CS vs. non‐CS
Baseline characteristics							
Maternal age (years)	25 ± 6^b^	25 ± 6	26 ± 6	0.51	25 ± 6	23 ± 5	<.001
Maternal years of education	4 ± 4	4 ± 3	4 ± 4	0.94	3.8 ± 3.5	5.2 ± 3.8	<.001
Maternal BMI (kg/m^a^)	22.0 ± 2.5	21.9 ± 2.7	22.0 ± 2.9	0.99	22.0 ± 2.7	22.6 ± 3.1	.012
Maternal Hb < 100 g/L	52/238 (22%)	49/239 (21%)	61/234 (26%)	0.34	162/711 (23%)	39/156 (25%)	.56
Maternal ZPP > 70 μmol/mol heme	38/238 (16%)	44/ 240 (18%)	44/233 (19%)	0.67	126/711 (18%)	30/157 (19%)	.69
Maternal sTfR > 6 mg/L	39/234 (17%)	38/236 (16%)	52/227 (23%)	0.14	129/697 (19%)	32/157 (20%)	.60
Mother HIV positive	33/238 (14%)^c^	26/239 (11%)	29/233 (12%)	0.61	88/710 (12%)	12/122 (10%)	.39
Gestational age at enrollment (week)	16.9 ± 2.0	16.9 ± 2.1	16.9 ± 2.1	0.98	16.9 ± 2.1	17.0 ± 2.1	.46
Primiparous	48/238 (20%)	53/239 (22%)	47/234 (20%)	0.82	148/711 (21%)	57/155 (37%)	<.001
Household asset index below median	111/236 (48%)	114/235 (49%)	120/232 (51%)	0.79	345/703 (49%)	32/114 (28%)	<.001
Site				0.96			<.001
Lungwena	125/238 (53%)	120/240 (50%)	122/234 (52%)		367/712 (51%)	32/157 (20%)	
Malindi	45/238 (19%)	47/240 (20%)	41/234 (18%)		133/712 (19%)	12/157 (8%)	
Mangochi	68/238 (29%)	73/240 (30%)	71/234 (30%)		212/712 (30%)	113/157 (72%)	

*Note*. IFA = iron/folic acid; MMN = multiple micronutrients; LNS = lipid‐based nutrient supplements; CS = cognitive sample; BMI = body mass index; Hb = hemoglobin; HIV = human immunodeficiency virus; sTfR = soluble transferrin receptor; ZPP = zinc protoporphyrin.

a *p*‐values were determined using analysis of variance for the continuous variables and Wald chi‐square for the categorical variables.

b Mean ± *SD* (all such values).

c N/total (%; all such values).

Participants successfully repeated an average of six digit sequences forward (*SD* = 2) and three (*SD* = 2) backward. In the category fluency test, the mean score was 32 (*SD* = 8) words produced in 60 s. The average mental rotation score was 63% (*SD* = 13) correct. In the functional health literacy test, the mean score was 16 (*SD* = 4) out of maximum 36 points, and the mean HOME score was 24 (*SD* = 4), also out of maximum 36. At enrollment, 23% of women were anemic (Hb < 100 g/L), 18% had low iron status based on elevated ZPP (>70 μmol/mol heme), and 19% had low iron status based on elevated sTfR (>6 mg/L).

Table 3 shows the mean maternal cognitive and HOME *z*‐scores in each intervention arm. The only score that was significantly different between intervention arms was the mental rotation *z*‐score. Women who received IFA scored 0.22 *SD* higher than women who received LNS, 95% CI [0.01, 0.44]; *p* = .040; adjusted *B* = 0.20; 95% CI [0.01, 0.39]; *p* = .032, where *B* is the unstandardized estimate of the regression coefficient, representing the change in *z*‐score of the outcome associated with a one‐unit change in the independent variable). Mental rotation *z*‐scores of women who received MMN did not differ from those who received IFA (unadjusted *B* = 0.05; 95% CI [−0.16, 0.27]; *p* = .83; adjusted *B* = 0.01; 95% CI [−0.17, 0.20]; *p* = .98) or LNS, though this difference was marginally significant in the adjusted analysis (unadjusted *B* = 0.17; 95% CI [−0.05, 0.39]; *p* = .15; adjusted *B* = 0.19; 95% CI [0.00, 0.38]; *p* = .054). In the per protocol analysis, only including the 498 women with >80% adherence to supplement consumption, the same pattern was found, with the only significant difference between intervention arms in the mental rotation *z*‐score (unadjusted *p =* .04 and adjusted *p* = .06), and women who received IFA scoring significantly higher than those who received LNS (unadjusted *B* = 0.25; 95% CI [0.01, 0.49]; *p* = .038; adjusted *B* = 0.18; 95% CI [−0.03, 0.40]; *p* = .11).

**Table 3:** Mean maternal cognitive and HOME *z*‐scores at the end of the intervention period^a^

	IFA	MMN	LNS	Unadjusted *p*‐value	Covariate‐adjusted *p*‐value	
	Mean ± *SD*	Mean ± *SD*	Mean ± *SD*			Covariates^b^
Digit span forward *z*‐score	−0.05 ± 1.01	0.04 ± 0.97	−0.01 ± 0.89	.61	.85	A, B, C, D, E, F, M, O
Digit span backward *z*‐score	−0.01 ± 0.94	−0.04 ± 0.99	0.01 ± 0.89	.84	.74	A, B, C, D, E, G, H
Category fluency *z*‐score	−0.03 ± 1.00	−0.04 ± 1.00	0.07 ± 1.00	.98	.59	A, B, D, G, H, I, J
Mental rotation *z*‐score	0.09 ± 1.00^a^	0.04 ± 0.99	−0.13 ± 1.01^b^	.040	.021	A, B, C, D, G, J, K, L, M
Functional health literacy *z*‐score	0.01 ± 0.99	0.02 ± 1.00	−0.03 ± 1.01	.89	.94	A, B, C, D, E, F, G, H, I, K, M
HOME *z*‐score	0.07 ± 1.03	0.02 ± 0.97	−0.08 ± 0.96	.28	.11	A, B, C, D, E, F, J, L, M, N

*Note*. IFA = iron/folic acid; MMN = multiple micronutrients; LNS = lipid‐based nutrient supplements; HOME = home observation for the measurement of the environment.

a Values are unadjusted mean ± SD or *p*‐value, estimated using analysis of variance/analysis of covariance. Values in a row with different superscript letters (a and b) are significantly different from each other.

b Covariates included data collector who administered the assessment for every outcome, all other covariates were collected at baseline. A = household asset index; B = maternal education; C = site; D = household food insecurity access scale; E = maternal body mass index; F = maternal mid‐upper arm circumference; G = maternal height; H = primiparity; I = maternal age; J = child age; K = maternal hemoglobin; L = maternal malaria positive rapid diagnostic test; M = season at enrollment; N = maternal HIV; O = gestational age at enrollment; P = child gender.

* *p* < .05 in adjusted model.

**Figure 2:**
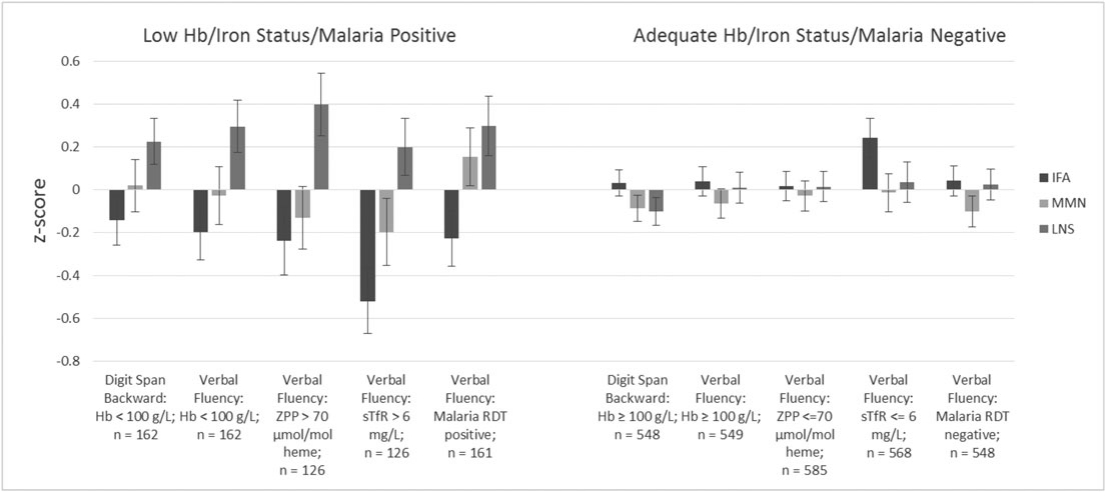
Mean cognitive *z*‐scores in each intervention group, stratified by significant effect modifiers. Error bars show the standard error of the mean. IFA = iron/folic acid; MMN = multiple micronutrients; LNS = lipid‐based nutrient supplements; Hb = hemoglobin; sTfR = soluble transferrin receptor; ZPP = zinc protoporphyrin; RDT = rapid diagnostic test

Of the 14 potential effect modifiers, four showed significant interactions with intervention arm for at least one outcome. The interaction between baseline maternal Hb and intervention arm was significant for two outcome scores (digit span backward and verbal fluency). For verbal fluency, the interaction was also significant between intervention arm and three effect modifiers: maternal ZPP, sTfR, and malaria. Figure 2 presents the mean cognitive *z*‐scores by intervention arm, stratified by all significant effect modifiers (for all other interaction terms tested, *p* > .05). Among women with low Hb, poor iron status, or malaria at enrollment (*n* = 126 to 162), women who received LNS scored 0.4 to 0.7 *SD* higher than those who received IFA in digit span backward and/or verbal fluency, 95% CI [0.06–0.29, 0.68–1.07], with scores of those who received MMN in between the other two arms. Among women with high Hb/iron status and malaria negative, differences between intervention arms were small (<0.2 *SD*).

Of the three nutritional biomarker components, the only component that was significantly associated with any score was the third component, representing higher breastmilk DHA and Vitamin B12 concentrations. Adjusting for baseline covariates, this component was associated with higher digit span forward (*B* = 0.12; 95% CI [0.02, 0.23]; *p* = .023) and verbal fluency scores (*B* = 0.15; 95% CI [0.04, 0.25]; *p* = .007). Additionally adjusting for intervention arm, both coefficients remained significant (digit span forward: *B* = 0.12; 95% CI [0.02, 0.23]; *p* = .025; verbal fluency: *B* = 0.14; 95% CI [0.03, 0.25]; *p* = .012).

## 4 DISCUSSION

We conducted a study of maternal cognition and caregiving in a randomized trial comparing three types of nutritional supplements provided to >700 women in Malawi over a 1‐year period from ≤20 weeks gestation to 6 months post‐partum. Regarding the first hypothesis that women who received MMN or LNS would score higher than the control group who received IFA, we found contrasting patterns for different cognitive abilities and groups of participants. In the full group of participants assessed for cognition, women who received IFA scored 0.22 *SD* higher than those who received LNS in visuospatial ability, equivalent to 3 IQ points. However, for verbal working memory and executive function, we found several significant effect modifiers showing a pattern of higher scores in the LNS arm compared to the IFA arm in certain subgroups. Among women with low Hb, poor iron status, or malaria at enrollment, those who received LNS scored 0.4 to 0.7 *SD* higher than those in the IFA arm in verbal fluency, equivalent to 6 to 10 IQ points, with the same pattern for digit span backward among anemic women. Regarding the second hypothesis that maternal scores would be associated with concurrent nutritional status, measured by biomarkers of iron, Vitamin A, B‐vitamins, and DHA, we found that breastmilk Vitamin B12 and DHA concentrations were associated with digit span forward and verbal fluency scores.

Given that iron plays a role in adult brain function in two primary ways, energy metabolism and monoamine neurotransmitter synthesis, uptake, and degradation (Beard & Connor, 2003), one potential explanation for the higher visuospatial scores in the IFA arm is the higher dose of iron (60 mg/day) they received during pregnancy compared to the MMN and LNS arms. However, in our study, the IFA arm did not score higher than the MMN arm, who received the same amount and duration of iron (20 mg/day until 6 months post‐partum) as the LNS arm, suggesting that the higher dose of iron during pregnancy may not account for this pattern. In addition, concurrent Hb/iron status was not associated with visuospatial scores. Moreover, the group differences in visuospatial ability were found 6 months beyond the point that IFA was exchanged for a placebo tablet. In animal models, cortical iron concentrations respond quickly to short periods of iron depletion and repletion, as short as 14 days (Pinero, Li, Connor, & Beard, 2000). Therefore, it is unlikely that effects would persist 6 months beyond the period of supplementation. Another potential explanation is that the higher amount of the omega‐6 fatty acid LA (4.6 g/day) compared to the omega‐3 fatty acid ALA (0.6 g/day) in LNS inhibited the synthesis of DHA (Harnack, Andersen, & Somoza, 2009), thus negatively affecting visuospatial performance. However, no effects of LNS supplementation were found on AA or DHA status, measured in plasma at 36 weeks gestation or in breastmilk at 6 months post‐partum (Oaks et al., 2017), suggesting that no such inhibition occurred. In addition, concurrent DHA status was not associated with visuospatial scores. It is also unlikely that the additional macrominerals in the LNS would negatively affect visuospatial ability; therefore, this finding may be due to chance.

The four subgroups of women for whom there was a positive association of LNS with working memory or executive function overlapped with each other. Whether iron deficiency was defined by high sTfR or ZPP at enrollment, about 40% of anemic women were iron deficient. Among anemic women, 38% were malaria positive, 13% were both iron deficient and malaria positive, and 35% were neither iron deficient nor malaria positive. In addition, 50% of iron‐deficient women and 60% of women who were malaria positive were not anemic. A potential explanation for the higher executive function and working memory scores among women with low Hb/iron status or malaria at enrollment who received LNS, compared to IFA, is the essential fatty acids, B‐vitamins, or Vitamin A contained in LNS. The association of DHA and Vitamin B12 status with verbal fluency suggests that these may be plausible mechanisms. The brain contains high levels of DHA, with the highest levels in the frontal cortex, which underlies verbal fluency performance. In animal models of ALA deficiency, the frontal cortex, along with the corpus striatum and pituitary gland, is the structure most severely affected, with an approximate 40% reduction of DHA; specifically, ALA deficiency results in disorders of monoaminergic neurotransmission in the frontal cortex (Bourre, 2004). Vitamin B12 is also involved in the synthesis of monoamine neurotransmitters (Leung & Kaplan, 2009), which play a role in executive function. Previous studies in elderly adults have reported associations between Vitamin B12 status and verbal fluency (Bourre, 2004; Robins Wahlin, Wahlin, Winblad, & Backman, 2001). As stated above, no effects of LNS supplementation were found on breastmilk concentrations of DHA (Oaks et al., 2017), but breastmilk Vitamin B12 concentration was higher in both the LNS and MMN groups compared to IFA (Haber‐Donohue, personal communication). However, the associations of DHA and Vitamin B12 with executive function and working memory were independent of supplementation with LNS, because adjusting for intervention arm did not reduce the magnitude of these associations. Therefore, these differences between intervention arms in specific subgroups may also be due to chance.

Strengths of this study were the randomized design, the large number of women assessed, and the assessment of nutritional biomarkers enabling examination of hypotheses regarding the mechanisms of the observed differences between intervention arms. A limitation was that we tested multiple outcomes and effect modifiers; therefore, some of the findings may be due to chance. Another limitation was that cognition was not assessed at baseline but only at endline; therefore, we were unable to analyze associations between changes in nutritional status and changes in cognition over the intervention period. In addition, we found several significant differences in baseline characteristics between participants who were assessed and those who were lost to follow‐up; therefore, the sample may not be representative of the participants in the larger study. Finally, the cognitive tests that we used were not computerized, and therefore, we were not able to capture small differences in response times; thus, our tests may not have been sensitive enough to detect small differences in cognitive performance.

For maternal functional health literacy and HOME scores, we did not find any differences between intervention arms or associations with nutrient status, overall or in any subgroups. Therefore, we did not find evidence that the observed differences in maternal cognition between intervention arms or associations with DHA and Vitamin B12 status translated into practical caregiving skills. This is consistent with a study in Bangladesh that did not find any positive effects of MMN compared to IFA on mother–infant interaction during a feeding episode (Frith et al., 2009). Rather, maternal education seemed to be the most important factor for health literacy and provision of nurturing care, as the strongest predictor of both of these scores. However, the HOME Inventory, which includes multiple items, some of which involve observation of mother–child interaction at a single home visit lasting about an hour, may not be sufficiently sensitive to detect small changes in maternal behavior. Other previous studies that found associations between maternal nutritional status and mother–child interaction used measures that involved more extensive observation and coding of maternal and child behavior (Kirksey et al., 1992; McCullough et al., 1990; Murray‐Kolb & Beard, 2009; Wachs et al., 1992).

Although previous trials examining the effect of nutritional supplementation during pregnancy and post‐partum on maternal cognition have focused on iron (Beard et al., 2005; Groner et al., 1986) or MMN (Prado et al., 2012), our finding of associations with DHA and Vitamin B12 status suggest that these are promising nutrients for future research to enhance attention and executive function during pregnancy and post‐partum. Consistent with previous studies that found effects of supplementation in undernourished and anemic women (Beard et al., 2005; Prado et al., 2012), we also found differences between intervention arms among these subgroups, suggesting that supplementation targeting women who are at risk for nutrient deficiencies is also a topic requiring further study.

## CONFLICTS OF INTEREST

The authors declare that they have no conflicts of interest.

## CONTRIBUTIONS

PA, UA, KGD, SAV, and KM designed the iLiNS‐DYAD‐M study; EP, UA, JP, and KGD designed the maternal cognition add‐on study; PA, UA, JS, JP, EP, and KM conducted field research; BMO led the analysis of biomarkers of fatty acid status; MH led the analysis of biomarkers of Vitamin A status; LA led the analysis of biomarkers of Vitamin B status; EP analyzed data and wrote the paper, with critical input and comments from all other authors; EP had primary responsibility for final content. All authors read and approved the final manuscript.

## Supporting information


**Table S1**. *Results of Principal Components Analysis of Hemoglobin and Nutritional Biomarkers*.
