# Supplementary material for: Associations of maternal nutrition during pregnancy and post‐partum with maternal cognition and caregiving
Source: Matern Child Nutr. 2017 Nov 2;14(2):e12546. doi: 10.1111/mcn.12546 (PMC5901033; doi:10.1111/mcn.12546)
Supplement: Supplementary file 1 — Table S1. Results of Principal Components Analysis of Hemoglobin and Nutritional Biomarkers. [file MCN-14-e12546-s001.docx]

# Online Supplemental Material for the Manuscript “**Associations of Maternal Nutrition during Pregnancy and Postpartum with Maternal Cognition and Caregiving**”

**Supplemental Methods**

***Assessment of Biomarkers of Nutritional Status***

Women expressed a full milk sample from a single breast during a home visit. A trained field worker then mixed the breast milk and collected a 10 mL sample, with the remaining milk provided to the infant by spoon. All samples were stored at -20°C within 24 h of collection and moved to -80°C for longer term storage. To assess DHA (% of total fatty acids by weight), breast milk was added to a mixture of solvents (methanol containing 14% boron trifluoride: toluene: methanol; 35:30:35 v/v/v, all from Sigma-Aldrich, St. Louis, MO). The tube was vortexed and heated in a hot bath at 100˚C for 45 minutes. After cooling, hexane (EMD Chemicals, USA) and distilled water were added. The sample was vortexed and centrifuged, and then an aliquot of the hexane phase was analyzed by gas chromatography using a GC-2010 (Shimadzu Corporation, Columbia, MD) equipped with a SP-2560, 100-m fused silica capillary column (0.25 mm internal diameter, 0.2 um film thickness; Supelco, Bellefonte, PA). Fatty acid composition was expressed as a percent by weight (wt%) of total identified fatty acids.

Riboflavin, flavin adenine dinucleotide, nicotinamide, and pyridoxal were analyzed as previously described (1) with a few modifications. Samples were analyzed after protein precipitation and removal of non-polar constituents with methyl-tert.-butyl ester (MTBE) using a Waters Alliance 2695 HPLC coupled to a Micromass Micro Quattro mass spectrometer (Waters, Milford, MA). Analytes were separated using a Waters Atlantis T3 (3µm, 2.1mm x 75mm) column guarded by a Thermo Scientific BDS-hypersil C18 Javelin guard (3µm, 3mm x 20mm) held at 40°C.

References

1. Hampel D, York ER, Allen LH. Ultra-performance liquid chromatography tandem mass-spectrometry (UPLC-MS/MS) for the rapid, simultaneous analysis of thiamin, riboflavin, flavin adenine dinucleotide, nicotinamide and pyridoxal in human milk. J Chromatogr B Analyt Technol Biomed Life Sci. 2012;903:7-13.

***Supplemental Table 1***. *Results of Principal Components Analysis of Hemoglobin and Nutritional Biomarkers.*

|  | Eigenvectors | | |
| --- | --- | --- | --- |
|  | Component 1 (Eigenvalue 1.6) | Component 2 (Eigenvalue 1.3) | Component 3  (Eigenvalue 1.2) |
| Hemoglobin (Hb) | **0.35** | **-0.31** | -0.26 |
| Zinc protoporphyrin (ZPP)* | **-0.36** | **0.42** | 0.30 |
| Plasma retinol | 0.18 | **-0.35** | -0.17 |
| Breastmilk DHA | 0.05 | -0.10 | **0.51** |
| Breastmilk vitamin B1 | 0.19 | **0.57** | -0.20 |
| Breastmilk vitamin B2 | **0.50** | 0.30 | 0.01 |
| Breastmilk vitamin B3 | **0.49** | -0.14 | 0.29 |
| Breastmilk vitamin B6 | **0.31** | **0.39** | -0.29 |
| Breastmilk vitamin B12 | **0.31** | 0.08 | **0.59** |

*Higher ZPP indicates poorer iron status

The three components explained 50% of variance in the nine hemoglobin and nutritional biomarker variables.
